# Immunomodulatory effects of Purion processed human amniotic membrane allografts in vitro

**DOI:** 10.1186/s12950-025-00477-3

**Published:** 2025-12-16

**Authors:** Sarah E. Moreno, Joseph Mikulin, Michelle Massee, Jimmie Lang, Tyler Olender, John R. Harper

**Affiliations:** 1 MiMedx Group, Inc., 1775 West Oak Commons Court NE, Marietta, 30062 Georgia; 2 HemoGenix, Inc., 1485 Garden of the Gods Rd STE 152, Colorado Springs, CO 80907 USA

**Keywords:** Amniotic membrane, Inflammation, Macrophage, Chronic wound, Acute wound

## Abstract

**Background:**

The immune system plays a pivotal role in progressing an injury through the healing cascade. However, comorbidities often lead to dysregulation of this response and are implicated in wound chronicity or stalling in the inflammatory phase, necessitating clinical intervention. The maternal-fetal interface, one of the most striking immunomodulatory microenvironments to be found in mammals, may be leveraged therapeutically in wound healing through the application of amniotic tissue allografts.

**Methods:**

This study investigates the influence of dehydrated human amnion chorion membrane (DHACM) and lyophilized human amnion and chorion membrane (LHACM) on the inflammatory response of monocytes and macrophages in vitro. Human THP-1 monocytes and macrophages were challenged with lipopolysaccharide (LPS) or LPS + interferon gamma (INFγ), respectively, to model inflammatory conditions.

**Results:**

LHACM and DHACM treatment significantly dampened inflammasome activity and pro-inflammatory protein production while enhancing cell survival in LPS-challenged monocytes. LPS/INFγ-challenged macrophages exhibited a phenotypic shift with treatment, synonymous with repair or regeneration functionality. This was further confirmed when these cells demonstrated corresponding attenuation of pro-inflammatory cytokine production, dampened inflammasome activity, and increased survival. Additionally, the rate of efferocytosis by DHACM and LHACM-treated macrophages was substantially elevated, indicating more efficient clearance of dead cell debris.

**Conclusion:**

These results indicate that DHACM and LHACM modulate the pro-inflammatory response of monocytes and macrophages, while enhancing the pro-reparative functions including efferocytotic capacity and cell survival. These data are suggestive of a potential cellular mechanism by which DHACM and LHACM may facilitate an efficient and appropriate inflammatory response to support the progression through the healing cascade.

## Background

The tissue repair process is a well-orchestrated cascade of sequential, yet over-lapping, events that are coordinated through the crosstalk of tissue-resident and infiltrating cell types. This communication facilitates resolution of one phase and transitions to the next until the wound has successfully navigated hemostasis, inflammation, proliferation and remodeling [1]. The chronicity of wounds can be attributed to severe injury, persistence of microbial contamination, and underlying comorbidities that have a direct impact on the behavior of key inflammatory cell types including monocytes and macrophages [2, 3]. In chronic wounds, these cells tend to enter a self-perpetuating inflammatory state that persists until interrupted by interventions such as proper wound care and advanced therapeutics. Targeting and correcting the proximate cellular and molecular causes of prolonged inflammation may be an effective method to resolve recalcitrant wounds [4].

During tissue repair, circulating monocytes selectively traffic to sites of injury and, particularly in the early phases of wound healing, produce inflammatory cytokines, and contribute to the local and systemic inflammation. Monocytes are highly infiltrative and can differentiate into longer lived and more phenotypically and functionally dynamic macrophages [5]. Traditionally, macrophage phenotypes have been over-simplified into pro-inflammatory (M1) and pro-repair/anti-inflammatory (M2) phenotypes [6]. However, the plasticity of monocytes and macrophages allows their adaptation to different microenvironments in which these cells assume distinct roles to create a phenotypic continuum that evolves throughout healing [4, 7–12]. For the purposes of this study, M1-like and M2-like will be used to indicate where on the macrophage spectrum these cell types fall. M1-like macrophages instigate early inflammation and are highly phagocytic to remove bacteria-filled neutrophils, damaged matrix, foreign debris, and bacteria which have hitherto evaded phagocytosis [13]. As the wound bed is cleared of complicating debris and pathogens, the population of macrophages transitions to an M2-like phenotype, secreting growth factors, chemokines, and cytokines to control and eventually resolve inflammation and initiate the next phase of healing [14].

The failure of macrophages to transition to an M2-like phenotype in a wound leads to a perpetual state of inflammation wherein M1-like macrophages in the wound bed produce concentrations of pro-inflammatory effector and signaling molecules at levels that prevent the orderly sequence of the wound healing cascade [15]. Monocytes infiltrating the wound from circulation are primed by this M1-dominant environment and preferentially differentiate into a pro-inflammatory macrophage phenotype. This feedback loop establishes a stern inflammatory microenvironment in the wound, leading to the continued recruitment of immune cells and excessive proteolytic activity over time, resulting in the destruction of the extracellular matrix [16]. Although the primary function of M1-like macrophages is to quickly and efficiently clear or sterilize the wound bed, chronic wounds are often overrun with bacteria and cellular debris. This suggests that persistent pro-inflammatory bias is not solely responsible for wound chronicity, and that exhaustion, senescence, poor viability, and suboptimal effector function are also contributing factors.

Current treatments for impaired wound healing focus mainly on optimization of controllable healing factors, e.g., clearance of infection, mechanical protection, and nutritional support. The few targeted approaches developed to date, including mainly topical application of growth factors, unfortunately demonstrate limited clinical efficacy [17]. Controlling the phenotypic balance between the pro-inflammatory M1-like to the pro-repair M2-like macrophage phenotypes in the wound has become an active area of research and may be an effective method to recalibrate the wound towards healing. Currently, there are few clinically available solutions for wound healing capable of addressing macrophage dysfunction that are absent of immunological side effects [14, 18, 19]. Amniotic membrane allografts have been widely used clinically in multiple applications and have been proven to be safe and effective [20–27]. In this study, two PURION^®^ processed amniotic tissue allografts were evaluated for their ability to modulate the inflammatory response of human monocytes and macrophages in conditions simulating a chronic wound. Lyophilized human amnion, intermediate layer, and chorion membrane (LHACM) is a freeze-dried tri-layer allograft, whereas, dehydrated human amnion, chorion membrane (DHACM) is an air-dried bi-layer allograft. Previous experiments have demonstrated this proprietary process retains well known regulatory proteins and sufficiently preserves their bioactivity to stimulate cellular activities across multiple cell types [28–34]. Additionally, DHACM has been shown to regulate inflammation through the neutralization of proinflammatory cytokines and proteases in an in vitro model of tendon injury [35]. This study aimed to evaluate the effect of LHACM and DHACM treatment on monocytes and macrophages to further elucidate how amniotic tissue allografts support the healing cascade in both acute and chronic wounds.

## Methods

### Human amnion and chorion membrane grafts

Birth tissue was donated under informed consent, following Caesarean sections, in compliance with the Food and Drug Administration’s (FDA) Good Tissue Practice and American Association of Tissue Banks (AATB) standards. All donors were tested and confirmed to be free of infectious diseases, including human immunodeficiency virus (HIV), human T-lymphotropic virus (HTLV), hepatitis B and C, and syphilis. Using aseptic techniques, the amniotic sac was separated from the placenta and processed in accordance with the proprietary PURION^®^ Process. For DHACM, the intermediate layer was mechanically removed from either side of the amnion and chorion layer, followed by air-dehydration at room temperature under controlled conditions. For LHACM, the intermediate layer was left intact, and the layers laminated such that the intermediate layer was sandwiched between the amnion and chorion prior to lyophilization.

### Extract preparation

Soluble extracts for cell culture experiments were prepared from DHACM and LHACM. Grafts from individual donors were minced and extracted at 4 °C overnight at 40 milligrams of tissue per milliliter of basal RPMI. The resulting extract was clarified and collected in a sterile container, followed by preparation at testing concentrations by dilution in basal RPMI. Appropriate concentrations were determined by studies to ensure the dose range encompassed the full effect of treatment (date not shown). Three independent extracts of DHACM and LHACM donors were used in each subsequent experiment.

Cell Culture and Differentiation.

THP-1 monocytes (ATCC TIB-202™) were cultured in complete medium, consisting of RPMI 1640 medium with L-Glutamine (basal RPMI) (Gibco) supplemented with 10% fetal bovine serum (FBS), 100 units/mL of penicillin, and 100 µg/mL of streptomycin (RPMI complete) at 37 °C/5% CO_2_ under humidified conditions. For passaging, cells were maintained at concentrations between 3 × 10^5^ cells/mL and 8 × 10^5^ cells/mL. For differentiation, THP-1 monocytes were seeded at a density of 5 × 10^5^ cells/mL and treated with 10 ng/mL phorbol 12-myristate 13-acetate (PMA) (Sigma-Aldrich) for 24 h, followed by washing and a 72-hour resting period in RPMI complete. After 72 h, the resulting macrophages (M0) were washed, harvested, counted, and re-plated per assay conditions. Jurkat cells (ATCC CRL-2899™) were maintained in RPMI 1640 medium with L-Glutamine (basal RPMI) (Gibco) supplemented with 10% fetal bovine serum (FBS), 100 units/mL of penicillin, and 100 µg/mL of streptomycin (RPMI complete) at 37 °C/5% CO_2_ under humidified conditions.

### Cell stimulation and polarization

THP-1 monocytes were seeded into 96 well plate at 1 × 10^5^ cells/well in RPMI complete and stimulated with 100 ng/mL LPS ± DHACM or LHACM extract (20, 10, and 1 mg/mL) for 24 h. After 24 h, monocytes were centrifuged at 400 x g for 5 minutes and supernatants were harvested and filtered to remove cellular debris. Monocyte pellets were resuspended in FACS buffer (PBS, 2% FBS, 2 mM EDTA) for flow cytometry analysis.

Differentiated THP-1 macrophages (M0) were seeded in 96 well plate at 1 × 10^5^ cells/well for stimulation. M0 macrophages were incubated for *24 h* with RPMI complete or the following sets of treatments: 100 ng/mL LPS and 20 ng/mL IFNγ (M_(IFNγ + LPS)_), or 20 mg/mL, 10 mg/mL, 1 mg/mL LHACM or DHACM in the presence or absence LPS +Nγ. Aft IFer 24 h, supernatants were collected and clarified. The resulting macrophages were harvested by the addition of Accutase (Stem Cell Technologies) for 15 min at 37 °C.

### Flow cytometry analysis

**Fig. 1:**
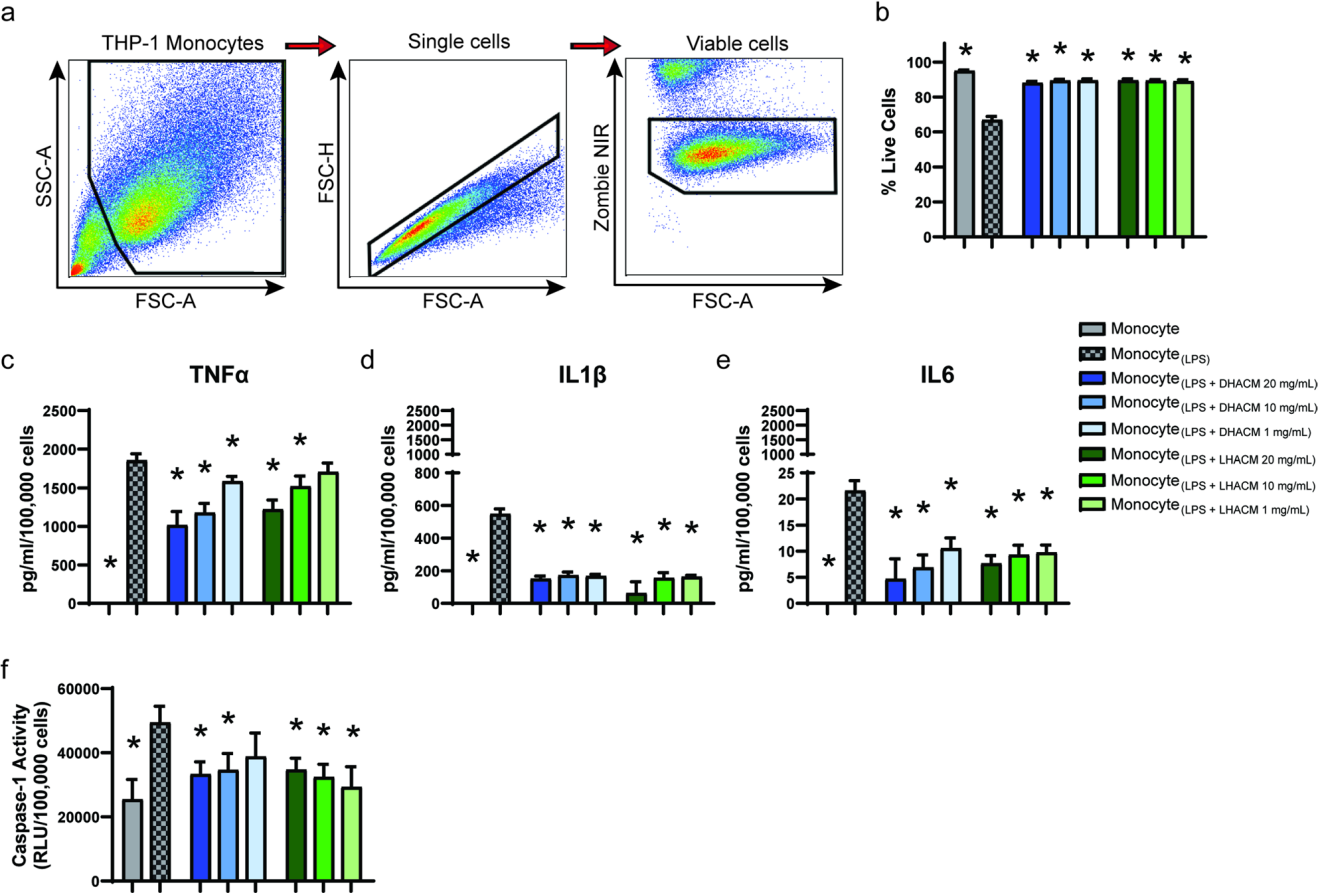
Evaluation of the effect of DHACM and LHACM on the pro-inflammatory response of THP-1 monocytes. THP-1 monocytes were treated with 100 ng/mL LPS +/- DHACM or LHACM for 24 h. (**a**) Example gating strategy to identify viable macrophages. THP-1 monocytes were gated on a forward scatter area × side scatter area pseudocolor plot to exclude debris. Single cells were gated using a forward scatter area × forward scatter height pseudocolor plot to exclude doublets. Resulting cells were gated again for detection of viable monocytes by means of Zombie NIR viability stain. The frequency of viable cells in the single cell gate (**b**) and production of proinflammatory cytokines (**c**) TNFα, (**d**) IL1β, and (**e**) IL6 were measured post-stimulation. (**f**) Inflammasome activation was assessed via the proteolytic activity of caspase-1 in the supernatant of monocytes stimulated with LPS ± DHACM or LHACM. Statistical analysis was performed using a one-way ANOVA followed by the Tukey’s test. * *p* < 0.05 indicates a statistically significant difference between treatment groups and Monocyte_(LPS)_

To assess viability of monocytes, cells were washed twice with FACS buffer, followed by centrifugation at 400 x g for 5 min. Cells were stained for 15 min at 4 °C in the dark with PBS containing Zombie NIR viability dye (BioLegend), washed twice in PBS, resuspended in 4% formaldehyde in PBS, and incubated at room temperature for 20 min in the dark to fix the cells. Cells were then washed and resuspended in FACS buffer. The fluorescence signal was acquired utilizing a spectral flow cytometer (Cytek Northern Lights, Cytek; Fremont, CA) and analyzed using FlowJo (version 10.10). Monocyte gating strategy is shown in Fig. 1a.

**Fig. 2:**
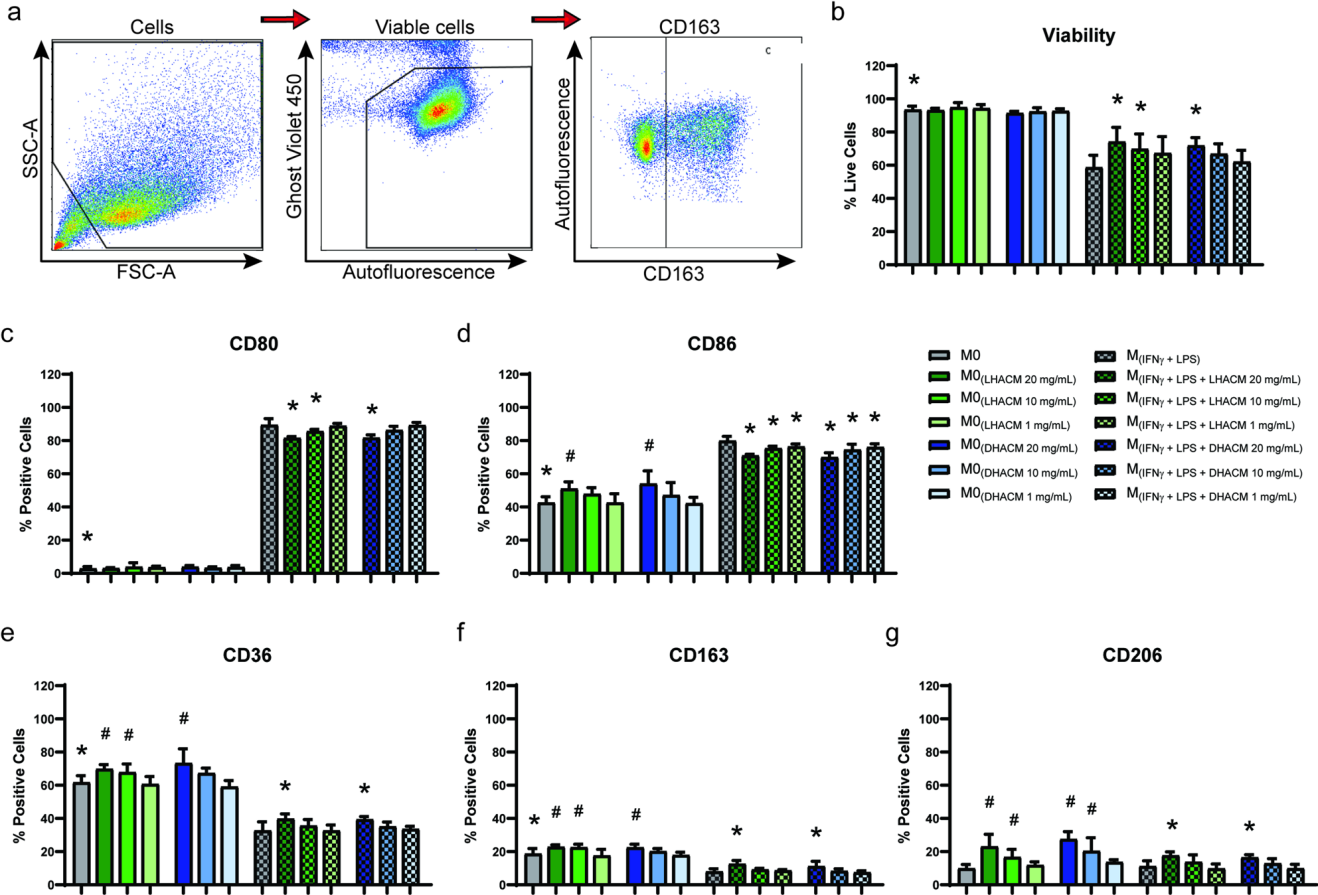
DHACM and LHACM modify macrophage polarization. Macrophages were generated by treating THP-1 monocytes with 10 ng/mL PMA for 24 h. Following differentiation, macrophages were polarized for 24 h with 100 ng/mL LPS and 20 ng/mL IFNγ, ± 20, 10, or 1 mg/mL DHACM or LHACM. (**a**) Example gating strategy to identify macrophages. Cells were gated on a forward scatter area × side scatter area pseudocolor plot. Resulting cells were gated again for detection of viable macrophages by means of Ghost Violet 450 viability stain, and within the viable macrophage population the surface markers were used to distinguish macrophage populations. The flow cytometry analysis assessed (**b**) the viability of resulting macrophages, the expression of key pro-inflammatory macrophage markers (**c**) CD80, (**d**) CD86, and pro-repair markers (**e**) CD36, and (**f**) CD163, and (**g**) CD206. * *p* ≤ 0.05 vs. M_(IFNγ + LPS);_ # *p* ≤ 0.05 vs. M0 based on a one-way ANOVA with a Tukey test

For polarization experiments, macrophages were stained for 15 min at 4 °C in the dark with FACS buffer containing F_c_ Receptor Binding Inhibitor Antibody (eBioscience) and Ghost Dye Violet 450 viability dye (Tonbo Biosciences). After 15 min, cells were stained in accordance with manufacturer’s instructions with antibodies against the following cell surface markers: pro-inflammatory markers CD80 (BV711), CD86 (PE-Cy5), and pro-repair markers CD163 (BV785), CD36 (AF700), and CD206 (PE-Dazzle 594) (BioLegend). Samples were then incubated for 30 min at 4 °C in the dark, washed twice with FACS buffer, resuspended in 4% formaldehyde in PBS, and incubated at room temperature for 20 min in the dark to fix the cells. Cells were then washed twice and resuspended in FACS buffer. Fluorescence was then acquired using spectral flow cytometry (Cytek Northern Lights, Cytek; Fremont, CA) and analyzed using FlowJo (version 10.10). Example gating strategy is shown in Fig. 2a.

### Evaluation of cell culture supernatant

The evaluation of inflammatory protein content was conducted using Luminex multiplex assays for IL1β, TNFα, and IL6. The assay was performed according to the manufacturer’s instructions, and each sample was tested in duplicate. Results were reported after subtracting the contribution from the extract alone.

Assessment of inflammasome activation was performed by measuring the proteolytic activity of caspase-1 using Caspase-Glo^®^ 1 inflammasome assays (Promega) according to manufacturer’s instructions. Data analysis and visualization were performed using GraphPad Prism. Both pro-inflammatory protein production and inflammasome activation from each sample were normalized to the viable cell population calculated by flow cytometric analysis of the corresponding cell pellets.

### Efferocytosis assay

Resting THP-1 macrophages (M0) were seeded into 96 well plates at 5 × 10^4^ cells/well and incubated for 48 h at 37 °C/5% CO_2_ with RPMI complete ± 20 mg/mL, 10 mg/mL or 1 mg/mL DHACM or LHACM. Efferocytosis assays were performed using an Efferocytosis Assay Kit (Cayman Chemical) according to the manufacturer’s protocol. Briefly, Jurkat cells were harvested and washed twice by centrifugation at 400 x g for 5 min. Cells were then resuspended in 2 mL of Cell-Based Assay Buffer, counted, and adjusted to a concentration of 1 × 10^7^ cells/mL. For staining of Jurkat bait cells, an equal volume of Cell-Based Assay Buffer containing CFSE was added to cells and incubated in the dark at 37 °C for 30 min. Cells were washed three times with RPMI complete, followed by resuspension in RPMI complete containing staurosporine and incubated at 37 °C in the dark for 6 h to induce apoptosis. The resulting CFSE-stained apoptotic bait cells were centrifuged at 300 x g for 5 min and washed twice with RPMI complete and resuspended in RPMI complete. Supernatant from macrophages was aspirated and replaced with RPMI complete containing bait cells at a 3:1 ratio of bait cells to macrophages. After an overnight incubation at 37 °C, supernatants were aspirated and macrophages were detached with Accutase treatment, transferred to 96 well V-bottom plates, and washed twice with FACS buffer. Flow cytometry was used to assess the efferocytotic load of the macrophages, which was analyzed and visualized using FlowJo and GraphPad Prism. Example of gating strategy is presented in Fig. 4a.

### Statistical analysis

Differences between groups were determined according to ANOVA. All analyses were performed using GraphPad Prism, version 10.0.2 (GraphPad Software, San Diego CA). Data are shown as average ± the SD. *P* ≤ 0.05 was considered statistically significant in all tests.

## Results

### DHACM and LHACM treatment modulates the monocyte inflammatory response

Monocytes recruited to a wound from the circulation can skew toward certain inflammatory functions upon sensing tissue damage, persisting without differentiating into macrophages and quickly exerting inflammatory pressure on the microenvironment the damaged tissue [36, 37]. To capture this initial inflammatory response, THP-1 monocytes were cultured for 24 h in non-stimulated conditions (Monocyte) or stimulated with LPS (Monocyte_(LPS)_). Monocyte_(LPS)_ exhibited a significant inflammatory response, resulting in increased pro-inflammatory cytokine production and a concomitantly sharp reduction in cell viability as compared to non-stimulated monocytes (Fig. 1). The treatment of Monocyte_(LPS)_ with soluble extracts of DHACM and LHACM in the presence of LPS prevented the loss of cellular viability observed in cells treated with LPS alone (Fig. 1b). Additionally, the increased production of inflammatory cytokines TNFα, IL1β, and IL6 was dampened with DHACM and LHACM treatment (Fig. 1c, d, e). TNFα levels were decreased at 20, 10, and 1 mg/mL DHACM whereas LHACM reduced TNFα levels at the 20 and 10 mg/mL concentrations (Fig. 1c). IL1β and IL6 were reduced by both DHACM and LHACM at all concentrations (Fig. 1d and 1). In line with a heightened inflammatory response, Monocyte_(LPS)_ increased the proteolytic activity of the inflammasome protease caspase-1, confirming activation of the inflammasome complex. DHACM at 20 and 10 mg/mL and LHACM at 20, 10, and 1 mg/mL both reduced caspase-1 activity in Monocyte_(LPS)_ supernatants (Fig. 1f).

### Macrophages treated with DHACM and LHACM exhibit phenotypic heterogeneity

To determine if DHACM and LHACM can influence the phenotypic fate of macrophages in vitro, M0 macrophages were treated with 20, 10, or 1 mg/mL DHACM or LHACM in the presence or absence of pro-inflammatory stimuli. Treatment with DHACM and LHACM alone did not impact the viability of M0 macrophages. However, in the presence of pro-inflammatory stimuli without DHACM or LHACM treatment, M_(IFNγ + LPS)_ cell viability dropped to 58%. Under pro-inflammatory stimuli with the addition of 20 mg/mL LHACM and DHACM, and 10 mg/mL LHACM cell viability never fell below 70% (Fig. 2b). Viability was unchanged with treatment at 1 mg/mL LHACM and DHACM. Surface expression of M1 macrophage-associated marker, CD86, was slightly elevated in M0 macrophages with both treatments at 20 mg/mL; however, this was a comparatively mild response when contrasted with that of the M_(IFNγ+LPS)_ group (Fig. 2d). Interestingly, the surface markers associated with pro-repair macrophages were also significantly elevated with DHACM and LHACM treatment compared to untreated M0 macrophages (Fig. 2e, f, g). Pro-inflammatory macrophages, M_(IFNγ + LPS)_, treated with DHACM and LHACM, in the continued presence of inflammatory cytokines, demonstrated a significant reduction in surface markers associated with the M1-like phenotype (Fig. 2c and d). Additionally, DHACM and LHACM increased the percentage of cells positive for expression of the scavenger receptors CD36 and CD163 and CD206, all of which are associated with M2-like phenotype of pro-repair macrophages (Fig. 2e, f, g).

### Regulation of pro-inflammatory response by DHACM and LHACM

**Fig. 3:**
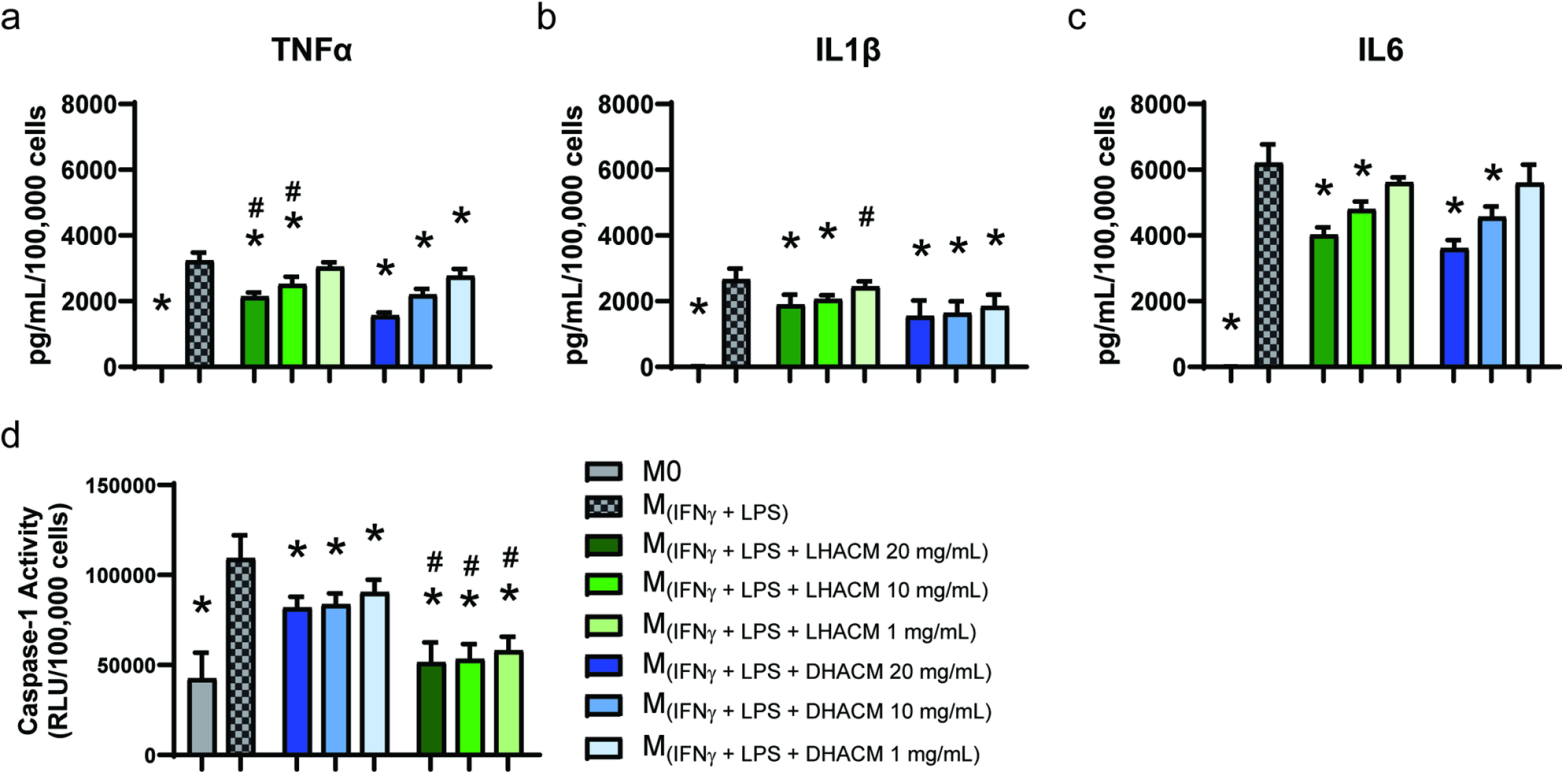
Treatment with DHACM and LHACM dampens THP-1 macrophage pro-inflammatory response. To induce an inflammatory response and inflammasome activity, THP-1 M0 macrophages were stimulated for 24 h with 100 ng/mL LPS and 20 ng/mL IFNγ, in the presence or absence of 20, 10, or 1 mg/mL DHACM or LHACM. Pro-inflammatory protein production of (**a**) TNFα, (**b**) IL1β, and (**c**) IL6 were measured following stimulation via Luminex analysis of cell supernatants and normalized to the number of live cells determined by flow cytometry. (**d**) Inflammasome function was determined by measuring the proteolytic activity of caspase-1 in the supernatant. * *p* ≤ 0.05 vs. M_(IFNγ + LPS);_ # *p* ≤ 0.05 vs. DHACM treatment at the same concentration based on a one-way ANOVA with a Tukey test

M_(IFNγ + LPS)_ macrophages demonstrated significantly increased production and secretion of pro-inflammatory cytokines TNFα, IL1β, and IL6 compared to M0 macrophages as expected (Fig. 3a, b and c). With the addition of both DHACM and LHACM treatment to M_(IFNγ + LPS)_ macrophages, a dose-dependent decrease in TNFα production was observed when compared to the untreated M_(IFNγ + LPS)_ macrophages (Fig. 3a). Increased production of IL1β was significantly blunted with DHACM treatment at all concentrations tested and by LHACM treatment at 20 and 10 mg/mL (Fig. 3b). Additionally, DHACM and LHACM treatment reduced the production of IL6 in a dose-dependent manner (Fig. 3c). Overall, both LHACM and DHACM regulated inflammatory cytokine secretion; however, differences were observed in the doses at which this effect was observed. Inflammatory macrophages (M_(IFNγ + LPS)_) predictably demonstrated increased inflammasome activation in response to stimulation. However, treatment with DHACM and LHACM reduced caspase-1 activity and therefore inflammasome activation in the supernatant of treated M_(IFNγ + LPS)_ cells (Fig. 2d). Interestingly, there was a significant difference in caspase-1 proteolytic activity between DHACM- and LHACM-treated conditions, indicating that LHACM can more effectively modulate inflammasome activity.

### DHACM and LHACM modulate the efferocytotic capacity of macrophages

**Fig. 4:**
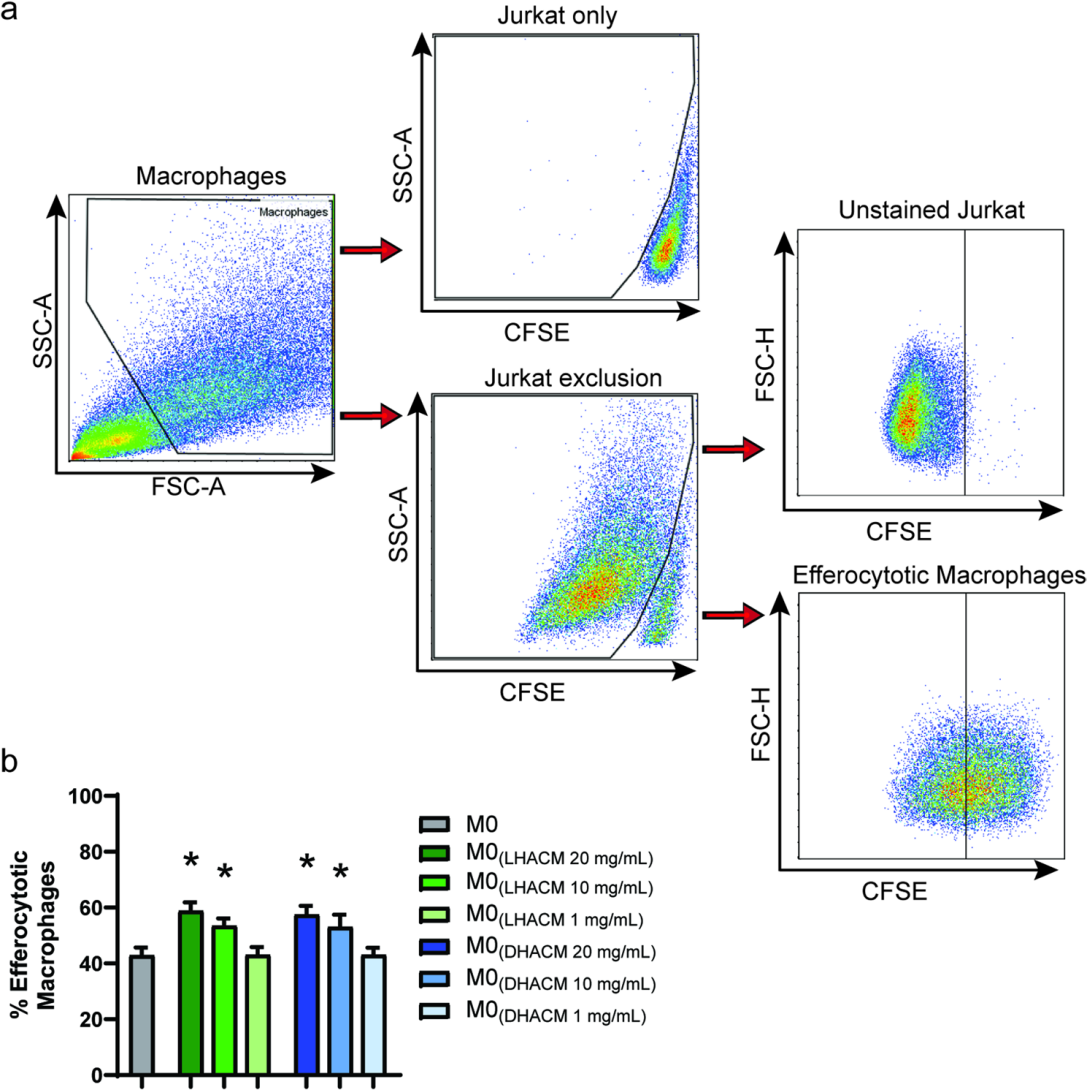
DHACM and LHACM modulate the efferocytotic function of macrophages in pro-inflammatory environment. (**a**) Gating strategy for the identification of efferocytotic macrophages. Representative flow cytometry plots showing the gating strategy. Macrophages were gated using a forward scatter area × side scatter area pseudocolor plot. Labeled Jurkat bait cells were excluded using a side scatter area × CFSE pseudocolor plot. Resulting cells were gated again for detection of efferocytotic macrophages by means forward scatter area × CFSE pseudocolor plots. (**b**) THP-1 M0 macrophages were treated with 20, 10, or 1 mg/mL DHACM or LHACM for 48 h before being incubated with CFSE-labeled apoptotic Jurkat cells. The frequency of Jurkat cell-containing macrophages was measured by flow cytometry. *P* ≤ 0.05 vs. M0 based on a one-way ANOVA with a Tukey test

Efferocytosis, the phagocytosis of apoptotic cells, is critical for resolution of inflammation and the restoration of tissue homeostasis, preventing secondary necrosis, elevated oxidative stress, ECM destruction, and opportunistic infection [38]. M0 macrophages treated with 20 mg/mL and 10 mg/mL DHACM and LHACM were subsequently more efferocytotic than untreated M0 cells as indicated by the increased frequency of CFSE- labeled bait cell-containing macrophages Jurkat (Fig. 4b). There was no difference in this effect between DHACM and LHACM.

## Discussion

Chronic inflammation occurs when acute inflammatory mechanisms fail to stabilize the wound environment and eliminate tissue injury [39]. Early treatments were developed to ameliorate inflammation altogether; however, these were found to be ineffective and even a further hinderance, as this immune response is a necessary component for proper tissue repair via transition along the wound healing cascade. Therefore, management of chronic wounds must include a balanced and more targeted approach to address the underlying causes of the dysregulated inflammation [14, 18, 40]. The clinical success of LHACM and DHACM products in the resolution of chronic wounds, including DFUs and VLUs, led to the hypothesis that these treatments may function in part as immunomodulators to overcome wound chronicity. Indeed, these data demonstrated the ability of DHACM and LHACM to alter the polarization state and activity of THP-1 monocytes and macrophages in favor of a more pro-repair phenotype in vitro. This was evident by altered cell surface markers, cytokine secretion, and functional outcomes.

Circulating monocytes recruited into wound sites differentiate into various subtypes of macrophages in the tissue according to the prevailing microenvironment, and play an indispensable part in wound healing by populating both inflammatory and reparative roles [41]. Therefore, monocytes are an attractive target for treating chronic inflammation due to their high levels of plasticity, entering from the bloodstream and changing their functional phenotype to ultimately supply both the M1-like and M2-like macrophage pools in the wound [4]. In pro-inflammatory environments, as modeled in this study by the treatment of THP-1 monocytes with LPS, the monocytes are stimulated to adopt an inflammatory phenotype with increased expression of inflammatory cytokines and activation of the inflammasome complex. Additionally, this pro-inflammatory environment significantly reduces the viability of these cells, as both the relevant effector proteins and the inflammasome cascade induce cell death over time. The presence of inflammatory monocytes will do little to resolve the underlying cause of inflammation and is in fact likely to perpetuate the cycle of inflammation. The introduction of DHACM and LHACM to monocytes treated with LPS tempered their inflammatory response, increasing cellular viability and changing the cytokine secretion profile. Treatment reduced the secretion of pro-inflammatory cytokines TNFα, IL1β, and IL6, which form the backbone of the pro-inflammatory response. Inflammasome activity was also down-regulated as evident by reduced caspase-1 proteolytic activity. The inflammasome is a multiprotein complex responsible for the caspase-1-dependent IL1β processing, and its assembly and activation depend upon the detection of pro-inflammatory signals [42, 43]. Active caspase-1 functions to cleave the proinflammatory IL1 family of cytokines into their bioactive forms, IL1β and IL18, and cause pyroptosis, a type of inflammatory cell death that may help restrict inflammatory activation of the cell but contributes pro-inflammatory cell debris to the wound environment over time [44]. Additionally, because IL1β is known to induce a pro-inflammatory macrophage phenotype, activation of the inflammasome may contribute to a positive feedback loop that sustains inflammation in chronic wounds and contributes to impaired healing [45]. Indeed, wounds from diabetic humans and mice have been found to contain elevated levels of IL1β and inflammasome activity [46–49]. DHACM and LHACM can impact monocyte biology and function and therefore may work by restoring the natural progression of wound healing.

As the monocytes mature into macrophages, receptors on the cell surface continue to sense environmental cues that influence the polarized state and corresponding cell functions [5]. Phenotypically pro-inflammatory M1-like macrophages express co-stimulatory molecules CD80 and CD86 and secrete inflammatory factors including TNFα, IL1β and IL6, allowing the macrophages to influence and coordinate with both nearby and distant immune cells. Pro-repair M2-like macrophages express CD206 [50, 51]. Interestingly, DHACM and LHACM favor a more blended macrophage phenotype. Naïve (M0) macrophages treated with LHACM or DHACM increase the surface expression of both pro-inflammatory and pro-repair markers with a more profound increase observed in pro-repair marker expression. This suggests that LHACM and DHACM treatment are not exclusively pro- or anti-inflammatory; rather, they promote a phenotype commensurate with the surrounding environment to drive progression through the healing cascade. This is further illustrated when LHACM and DHACM treatment were administered under pro-inflammatory conditions. Treatment decreased the surface marker expression of CD80 and CD86 while increasing the expression of pro-repair markers CD36, a primary efferocytotic receptor whose signaling promotes TGF [52, 53]. Not surprisingly, treatment also corresponded with reduced secretion of TNFα, IL1β, and IL6, as well as reduced inflammasome activity. Indeed, the differential impact of LHACM and DHACM suggests these treatments may modulate macrophage phenotypes to exhibit a mixed phenotype that would allow for a more controlled and balanced resolution to inflammation without compromising subsequent phases of the wound healing cascade.

Macrophages treated with LHACM and DHACM not only dampened the expression of pro-inflammatory M1-like cell surface markers but also induced those associated with repair. To determine whether this phenotypic adjustment led to a similar functional change, LHACM- and DHACM-treated macrophages were evaluated for their ability to clear cellular debris via a mechanism called efferocytosis. Generally, M2-like macrophages have higher efferocytosis capacity compared to that of M1-like macrophages [54]. Efferocytosis is an anti-inflammatory process that involves the removal of apoptotic cells and is integral to establishing and maintaining tissue homeostasis [55]. For example, macrophages isolated from murine diabetic wounds exhibit impaired efferocytosis that lead to an increase of apoptotic cells in the wound. This has been further verified in humans, as an increase in apoptotic cell load has been observed in wound tissue of individuals with diabetes [56, 57]. This is likely due to the prevalence of M1-like macrophages in chronic wounds and their inability to transition to an M2-like phenotype. Introduction of DHACM and LHACM treatment increased the expression of CD36 on the cell surface of M0 macrophages with and without the addition of pro-inflammatory stimuli. This increase in the expression of CD36, a receptor known to recognize the “eat me” ligands on apoptotic cells, is likely mechanistically linked to the increased efficiency with which the treated macrophages were able to clear apoptotic jurkat cells [58]. This may indicate that naive macrophages exposed to DHACM or LHACM treatment are more inclined to assume pro-reparative responsibilities within the wound to resolve excessive inflammation and prevent the accumulation of apoptotic cell debris.

Together these data support the hypothesis that DHACM and LHACM can regulate key cell types in the inflammatory cascade which may facilitate improved healing in complex wounds. These experiments were carried out on naive macrophages or under pro-inflammatory conditions and therefore the conclusions are largely limited to the effects of treatment on infiltrating monocytes and macrophages. In both scenarios, LHACM and DHACM treatment induce a blended macrophage phenotype with features associated with tissue repair. To better model the effects of treatment on established inflammation, it would be interesting in future studies to investigate the impact of LHACM and DHACM treatment on macrophages pre-treated with inflammatory stimulants. Additionally, these studies used THP-1 cells for the generation of macrophages. It is known that THP-1 macrophages do not retain the same plasticity nor spectrum of response to stimulation that PBMC-derived macrophages do, and often demonstrate important differences in surface marker expression and cellular function [59, 60]. Therefore, these foundational results should be confirmed in experiments utilizing PMBC-derived macrophages. Finally, there is always the possibility that in vitro results will fail to recapitulate in a more complex in vivo environment. Notwithstanding these limitations, this study illustrates the multifactorial immunomodulatory effect of LHACM and DHACM on monocyte and macrophage cells in vitro.

## Conclusion

**Fig. 5:**
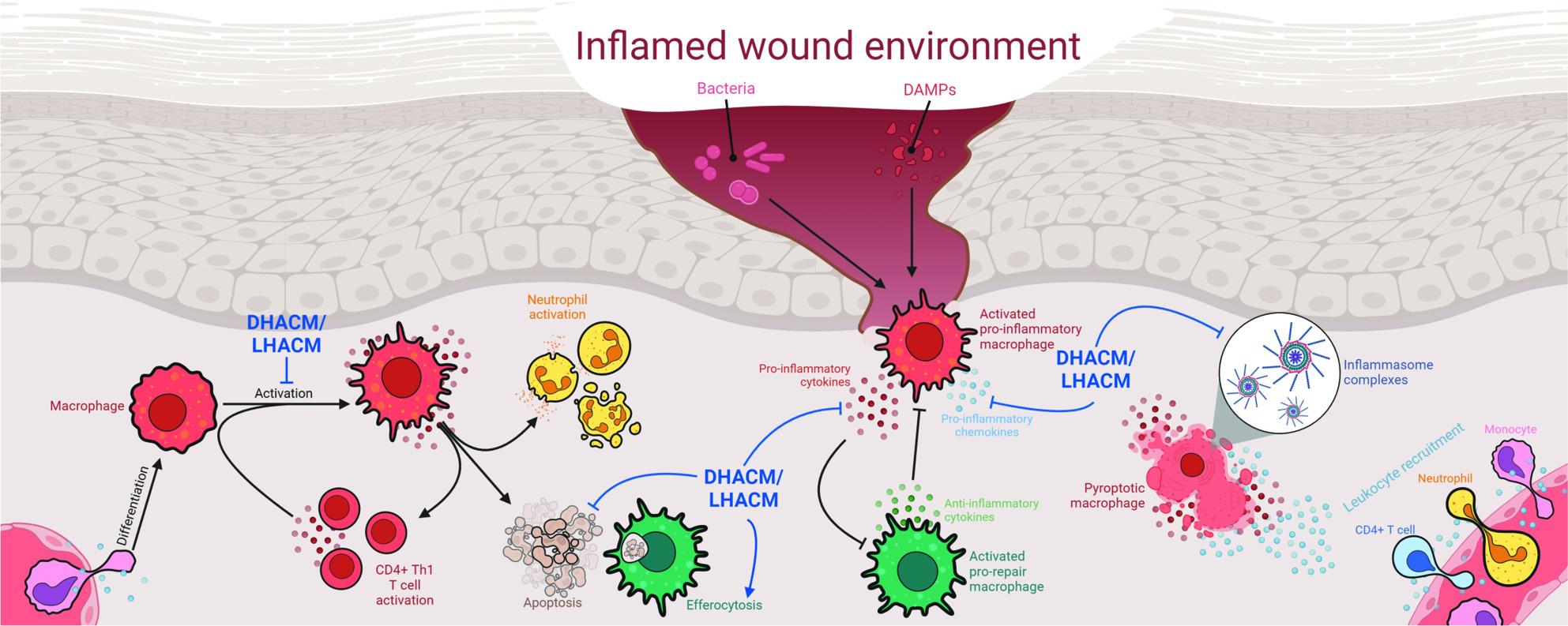
Summary of the multimodule effect of DHACM and LHACM on macrophage effector function in the wound. DHACM and LHACM modifies complex macrophage biology both in the absence and presence of inflammatory stimulus. Treatment blunts pro-inflammatory macrophage polarization and promotes a blended macrophage surface phenotype. During pro-inflammatory challenge, treatment protects macrophages from cell death, reduces the production of pro-inflammatory cytokines and chemokines, and dampens the activity of inflammasome effector protein caspase-1. DHACM and LHACM-elicited macrophages also demonstrate an increased rate of efferocytosis. Created using BioRender

This study identified a multifactorial effect of human amniotic membrane-derived allografts on inflammation (Fig. 5). These data demonstrate that LHACM and DHACM modulate significant features of inflammatory macrophage biology while enhancing pro-reparative functions including efferocytosis and cell survival. These distinct changes in macrophage phenotype and function provide insight into the potential mechanisms by which DHACM and LHACM may support the healing cascade and facilitate tissue repair.

## Data Availability

The datasets analyzed during the current study are available from the corresponding author upon request.
